# Smart pumps and random safety audits in a Neonatal Intensive Care Unit: a new challenge for patient safety

**DOI:** 10.1186/s12887-015-0521-6

**Published:** 2015-12-11

**Authors:** Elena Bergon-Sendin, Carmen Perez-Grande, David Lora-Pablos, María Teresa Moral-Pumarega, Ana Melgar-Bonis, Carmen Peña-Peloche, Mercedes Diezma-Rodino, Lidia García-San Jose, Esther Cabañes-Alonso, Carmen Rosa Pallas-Alonso

**Affiliations:** Department of Neonatology, Biomedical Research Institute i + 12, 12 de Octubre University Hospital, Avenida de Córdoba s/n, Madrid, 28041 Spain

**Keywords:** Smart pumps, Random safety audits, Technology, Patient safety, Adverse events, Neonatal Intensive Care Unit, Newborn

## Abstract

**Background:**

Random safety audits (RSA) are a safety tool enabling prevention of adverse events, but they have not been widely used in hospitals. The aim of this study was to use RSAs to assess and compare the frequency of appropriate use of infusion pump safety systems in a Neonatal Intensive Care Unit (NICU) before and after quality improvement interventions and to analyse the intravenous medication programming data.

**Methods:**

Prospective, observational study comparing the frequency of appropriate use of Alaris® CC smart pumps through RSAs over two periods, from 1 January to 31 December 2012 and from 1 November 2014 to 31 January 2015. *Appropriate use* was defined as all evaluated variables being correctly programmed into the same device. Between the two periods they were established interventions to improve the use of pumps. The information recorded at the pumps with the new security system, also extracted for one year.

**Results:**

Fifty-two measurements were collected during the first period and 160 measurements during the second period. The frequency of appropriate use was 73.13 % (117/160) in the second period versus 0 % (0/52) in the first period (*p* < 0.0001). Information was recorded on 44,924 infusions; in 46.03 % (20,680/44,924) of cases the drug name was recorded. In 2.5 % (532/20,680) of cases there was an attempt to exceed the absolute limit.

**Conclusions:**

Random Safety Audits were a very useful tool for detecting inappropriate use of pumps in the NICU. The improvement strategies were effective for improving appropriate use and programming of the intravenous medication infusion pumps in our NICU.

## Background

Advances in neonatology care have achieved an increase in the survival rate of premature and ill newborns. These patients frequently require intravenous treatment which poses a higher risk of adverse events [1]. The incidence of medication-related errors in children is two- to three-times higher than in adults. In addition, newborns are the most vulnerable patients as their internal reserves, which buffer the consequences of medication errors, are more limited [2-4].

Furthermore, Neonatal Intensive Care Units (NICUs) are highly complex units, not only because of the type of patients they care for, but also because of the wide range of technology they use. Multiple unplanned, critical situations occur in NICUs, which can lead to related events through the inappropriate use of technological devices. Healthcare technology is widely integrated into today’s intensive care units. However, the prevalence of device-related errors and their consequences for patients are still not well-defined [5, 6].

For decades, industries dealing with high-risk situations, such as aviation, have developed safety tools (e.g. checklists, root-cause analysis, failure mode and effects analysis, random safety audits) to decrease the possibility of human error and to detect system failures [6, 7]. Given that safety is an essential component of the quality of care for hospitalised patients, all possible measures should be used to try to reduce adverse events. However, Random Safety Audits (RSA), a much-used tool in industry due to their great ability to identify errors and situations of potential risk, are still little-used in the hospital environment. There is very little published data on their use as a tool for safety and quality control [8, 9]. An RSA consists of continuously monitoring procedures considered to be high risk in order to identify and address error-prone points in the system that are difficult to detect with other methods, and this before they cause patient harm. The application of this method in a hospital setting could be extremely valuable, as it evaluates clinical practice in real time and provides immediate feedback to the staff in the Unit [6, 7]. Moreover, this method put in place by frontline clinical staff, only requires simple training and involves a low cost of implementation.

In addition, technologies to administer intravenous medication which incorporate ever more advanced safety systems and new safety software are being developed and have demonstrated a positive impact on patient quality of care, decreasing medication-related adverse events. This also has a positive effect on healthcare personnel by improving work flow, reducing legal risk, and reducing costs [10].

The main purpose of RSAs is to continuously monitor certain procedures. However another possible use would be to consistently detect weaknesses and subsequently apply interventions. The RSAs could measure the impact of the interventions. In light of the scarce availability of information about medication infusion pumps in NICUs and the use of RSAs in hospitals, we established rounds of audits on the use of pump safety systems. Given the results, we designed quality improvement strategies. New rounds of audits were later established to test the efficacy of the interventions.

Thus, the objective of our study was to assess and compare the frequency of appropriate use of the infusion pump safety systems by using RSAs in a level III-C neonatal intensive care unit before and after an intervention to improve infusion pump use, as well as to analyse the intravenous programming data in our unit.

## Methods

Prospective, observational study comparing two periods through rounds of audits in which data related to the use of Alaris® CC syringe infusion pump safety systems was collected in a level III-C NICU with around 500 admissions per year in intensive care. Our NICU is divided into three areas for critical care – a large one with 10 beds and two small ones, one with 4 beds and the other with 5 beds – and two additional areas with 24 medium-care cots. The large area, NICU-A, is for full-term neonates, surgical problems and patients transferred from other hospitals. The other two areas, NICU-B and C, house babies up to 30 weeks of age. The patient/nurse ratio in the NICU is 2.1.

Physicians and nurses were surveyed according to the modified Delphi technique on the technological devices and procedures for which the recommendations for use were apparently often not met, ensuring that the most relevant equipment and procedures were included. This was a structured methodology in which, through a questionnaire and group meetings (doctors and nurses), a consensus was reached as to the resources and procedures apparently affected by protocol non-compliance. A total of 23 technological devices and procedures were selected and 23 cards were produced, each containing the variables to be evaluated for each device or procedure. One of the resources audited was the medication pumps.

In the first period (1 January 2012 to 31 December 2012) RSAs were performed on these 23 different resources and procedures, one of which was the medication pumps. The data from this first period was analysed in 2013, when it was discovered that the use of the pumps was inappropriate in most cases. The program was immediately installed and theoretical and practical training was provided in workshops for doctors and nurses. After a period of adaptation to the program, all the information stored in the pumps in 2014 (shown in Fig. 2) was downloaded. In the second period (1 November 2014 to 31 January 2015), there were more RSAs, in this case only affecting the medication pumps, so a large number of data were collected in only three months. The data presented correspond to the first and second period, plus the downloaded information stored in the pumps throughout 2014.

The first period for rounds of audits was from 1 January 2012 to 31 December 2012 (a calendar year); the objective during this round was to understand the baseline position in the Unit in relation to use of infusion pumps and other devices/procedures. During the study period, two days each week (normal working days or weekends/holidays) and the shift (early or late) were selected at random. Early shift was from 08:00 a.m. to 3:00 p.m. and late shift was from 3:00 p.m. to 10:00 p.m. The night shift was not audited as the investigators were not available. At the beginning of each week, a person unrelated to the system randomly drew two cards with the days of the week and shifts (early or late) and the RSAs to be audited. On each of these days, an investigator (EBS or MCPG) identified devices or procedures that were to be audited and that were in use on at least one patient, and audited all study variables for the selected procedures or equipment. The NICU staff did not know the purpose of the audit but, if an error was detected that might involve a potential danger to the patient, the caregivers were immediately informed. During this first 12-month period, the 23 resources and procedures were audited, so RSAs were only performed on the medication pumps when the card was randomly selected. When that occurred, all the pumps currently in use in all the neonates in the NICU were audited. In the case of the infusion pumps, the audited variables were: line type (central/peripheral), pressure alarm programmed (yes/no), appropriate pressure alarm (yes/no) (it was considered appropriate when programmed to 30–50 mmHg above the working pressure), volume to infuse programmed (yes/no), correct programming for volume to infuse (yes/no), correct infusion rate (yes/no). In addition, an outcome variable called *appropriate use* was defined. In this variable, the overall outcome was very demanding since the outcome *appropriate use* was only assigned when all the evaluated items were completely correct for a same device. In the following months all the data collected from the infusion pump audits were analysed and strategies to improve the way the pumps and their safety systems are used were planned. Firstly, these strategies consisted of updating the drug library and changing the software of all the unit’s pumps for others with better safety filters (Guardrails CQI Event Reporter®, CareFusion). This program allows predetermined relative and absolute drug infusion rate limits to be set. If the relative limits (both upper and lower) are breached, an alarm sounds, but the infusion is allowed to continue by confirming the program. If the absolute limit (only the upper limit) is breached, the alarm requires the infusion to be cancelled or the pump to be reprogrammed correctly. In addition, it is possible to collect prospective data automatically and to analyse data on intravenous drug infusion programming, which enabled us to analyse the intravenous medication programming data for 2014. In our unit these pumps are used for volume bolus infusions and platelet transfusions as well as for administering intravenous medication. Similarly, a low-pressure alarm was pre-established by default (60 mmHg), which the nurse could change as appropriate.

At the same time, theoretical training sessions and practical workshops on using the syringe pumps were given to all doctors and nurses in the Unit. Furthermore, a detailed written protocol was prepared on programming and using these pumps, accessible to all Unit personnel.

After these interventions, audits were performed again, evaluating only the infusion pumps over a 3-month period (1 November 2014 to 31 January 2015), to verify the efficacy of the strategy. Two days a week, selected at random, in a shift also selected at random, an RSA was performed on all the medication pumps currently in use in the NICU. As only the medication pumps were audited in this second period, a large number of data were collected, so collection was not continued for a full year. The degree of agreement between the two investigators was analysed by simultaneous rounds of audits.

### Other variables

Data was collected about the patient, time, and the characteristics of the place of admission to the NICU to assess if they influenced the use of the equipment’s safety mechanisms: birth weight, gestational age, sex, working day or weekend/holiday, morning or afternoon shift, NICU occupancy at the time of audit, and location of the patient within the unit. It was not necessary to request informed consent for the patients, since the use of infusion pumps on hospitalised patients is normal practice and the study did not involve any changes to the therapeutic treatment, the study object was the infusion devices and the information related to the patients was confidential (through a study code).

### Ethical issues

This study involved quality strategies for improving patient safety and thus did not require institutional Review Board approval. The objetive of the study is a service audit and no formal review is required by the Ethics Committee under current Spanish law. The study consent was obtained from the Head Doctor and the Head Nurses of the Unit.

### Analysis plan

Continuous variables are presented as mean ± SD and categorical variables as absolute and relative frequencies. The reproducibility of the observations made by the two study investigators was estimated with the kappa coefficient. The statistical significance of the comparison of proportions was determined using chi-squared or Fisher’s exact test from contingency tables. Comparisons of the distributions of ordinal and continuous measurements were made using the Wilcoxon–Mann–Whitney test or Student t-test, as appropriate. Logistic regression analysis was used to estimate the strength of correlation between appropriate use and several covariates such as gestational age, birth weight, sex, location in the NICU, working days and weekends/holidays, shift, month and occupancy. Results are presented as odds ratios and 95 % confidence intervals (CI).

## Results

### Random safety audits

During the first study period 10 rounds of audits were performed, which collected a total of 52 infusion pump measurements of a total of 32 patients. During the second study period 25 rounds of audits were performed that collected a total of 160 measurements (83 patients). The kappa coefficient of inter-observer agreement between the two investigators performing the audits was 0.93.

The results for the assessed variables are shown in Table 1. The frequency of appropriate pump use was 73.13 % (117/160) in the second period compared to 0 % (0/52) in the first period (*p* < 0.0001). During the first study period, the patient characteristics, time, and location in the unit did not influence the appropriate use of the infusion pumps (see Table 2). It was not possible to compare working days with weekends/holidays in the first period because by chance all the days on which the pumps were assessed were working days (during the first period, audits were carried out on 29 non-working days but, as 23 technological resources/procedures were audited at random, the audit card for the infusion pumps was not drawn on any of these non-working days).

**Table 1:** Frequency of assessed variables during the rounds of syringe infusion pump audits

Alaris® CC Syringe Pumps (N measurements)			N		Appropriate Use % (Confidence Interval)		
			Period 1 (N 52)	Period 2 (N 160)	Period 1	Period 2	P value
Line Type	Central		35	117	67.31 (54.56–80.05)	73.13 (66.26–79.99)	0.5275
Pressure alarm	Programmed	Yes	48	160	92.31 (85.07–99.55)	100 (97.72–100)	0.0031
	Programmed	Good	1	132	1.92 (1.81–5.65)	82.50 (76.61–88.39)	0.0001
VTI	Programmed	Yes	37	134	71.15 (58.84–83.46)	83.75 (78.03–89.47)	0.0725
	Programmed	Good	26	134	50 (36.41–63.59)	83.75 (78.03–89.47)	0.0001
Infusion rate	Good		51	160	98.08 (94.34–100)	100 (97.72–100)	0.5529
Appropriate use	Yes		0	117	0 (0–6.85)	73.13 (66.26–79.99)	<0.0001

VTI: volume to infuse in a certain time

**Table 2:** First study period: analysis of factors that could influence the studied infusion pump variables

Period 1 (N measurements)	Birth weight (g)			Gestational age (w)			Sex			Shift			Location		
	(N 51)*			(N 52)			(N 52)			(N 52)			(N 52)		
	<1500 (15/51)*	≥1500 (36/51)*	p	<32 (19/52)	≥32 (33/52)	p	Male (27/52)	Female (25/52)	p	Morning (17/52)	Afternoon (35/52)	p	Large area (35/52)	Small area (14/52)	p
Type of line = central % (N 35)	46.67 (7/15)*	75 (27/36)*	0.01	47.37 (9/19)	78.79 (26/33)	0.03	66.67 (18/27)	68 (17/25)	1	76.47 (13/17)	62.86 (22/35)	0.36	78.95 (30/38)	35.71 (5/14)	0.06
% Pressure alarm programmed (N 48)	93.33 (14/15)*	91.67 (33/36)*	1	94.74 (18/19)	90.91 (30/33)	1	96.30 (26/27)	88 (22/25)	0.34	100 (17/17)	88.57 (31/35)	0.29	92.11 (35/38)	92.86 (13/14)	1
% Pressure alarm appropriate (N 1)	0 (0/15)*	1.96 (1/36)*	1	0 (0/19)	3.03 (1/33)	1	3.70 (1/27)	0 (0/25)	1	0 (0/17)	2.86 (1/35)	1	2.63 (1/38)	0 (0/14)	1
% VTI programmed (N 37)	80 (12/15)*	66.67 (24/36)*	0.5	78.95 (15/19)	66.67 (22/33)	0.52	74.07 (20/27)	68 (17/25)	0.76	64.71 (11/17)	74.29 (26/35)	0.52	65.79 (25/38)	85.71 (12/14)	0.3
% VTI appropriate (N 26)	66.67 (10/15)*	44.44 (16/36)*	0.22	57.89 (11/19)	45.45 (15/33)	0.56	51.85 (14/27)	48 (12/25)	1	35.29 (6/17)	57.14 (20/35)	0.23	44.74 (17/38)	64.29 (9/14)	0.34
% Infusion rate appropriate (N 51)	100 (15/15)*	97.22 (35/36)*	1	100 (19/19)	96.97 (32/33)	1	96.30 (26/27)	100 (25/25)	1	100 (17/17)	97.14 (34/35)	1	97.37 (37/38)	100 (14/14)	1
% Appropriate use (N 52)	0 (0/15)*	0 (0/36)*	-	0 (0/19)	0 (0/33)	-	0 (0/27)	0 (0/25)	-	0 (0/17)	0 (0/35)	-	0 (0/38)	0 (0/14)	-

* One value lost. VTI: volume to infuse in a certain time

During the second study period, the frequency of appropriate use of infusion pumps was significantly higher in the small area 82.28 % (65/79) versus 64.20 % (52/81) in the large area (*p* < 0.01) (Table 3). Patient characteristics, time, and unit occupancy did not influence the appropriate use of the infusion pumps.

**Table 3:** Second study period: analysis of factors that could influence the studied infusion pump variables

Period 2 (N measurements)	Birth weight (g)			Gestational age (w)			Sex			Day			Shift			Placement		
	(N 160)			(N 160)			(N 160)			(N 160)			(N 160)			(N 160)		
	<1500 (114/160)	≥1500 (46/160)	p	<32 (116/160)	≥32 (44/160)	p	Male (91/160)	Female (69/160)	p	Work day (105/160)	Holiday (55/160)	p	Morning (67/160)	Afternoon (93/160)	p	Large box (81/160)	Small box (79/160)	p
Type of line = central % (N 117)	72.81 (83/114)	73.91 (34/46)	1	73.28 (85/116)	72.73 (32/44)	1	63.74 (58/91)	85.51 (59/69)	0.002	74.29 (78/105)	70.91 (39/55)	0.7	68.66 (46/67)	76.43 (71/93)	0.28	62.96 (51/81)	83.54 (66/79)	0.004
% Pressure alarm programmed (N 160)	100 (114/114)	100 (46/46)	-	100 (116/116)	100 (44/44)	-	100 (91/91)	100 (69/69)	-	100 (105/105)	100 (55/55)	-	100 (67/67)	100 (93/93)	-	100 (81/81)	100 (79/79)	-
% Pressure alarm appropriate (N 132)	82.46 (94/114)	82.61 (38/46)	1	81.90 (95/116)	84.09 (37/44)	0.81	86.81 (79/91)	76.81 (53/69)	0.14	83.81 (88/105)	80 (44/55)	0.66	80.60 (54/67)	83.87 (78/93)	0.67	72.84 (59/81)	92.41 (73/79)	0.001
% VTI programmed (N 134)	85.09 (97/114)	80.43 (37/46)	0.48	84.48 (98/116)	81.82 (36/44)	0.81	91.21 (83/91)	73.91 (51/69)	0.004	80 (84/105)	90.91 (50/55)	0.11	83.58 (56/67)	83.87 (78/93)	1	85.19 (69/81)	82.28 (65/79)	0.67
% VTI appropriate (N 134)	85.09 (97/114)	80.43 (37/46)	0.48	84.48 (98/116)	81.82 (36/44)	0.81	91.21 (83/91)	73.91 (51/69)	0.004	80 (84/105)	90.91 (50/55)	0.11	83.58 (56/67)	83.87 (78/93)	1	85.19 (69/81)	82.28 (65/79)	0.67
% Infusion rate appropriate (N 160)	100 (114/114)	100 (46/46)	-	100 (116/116)	100 (44/44)	-	100 (91/91)	100 (69/69)	-	100 (105/105)	100 (55/55)	-	100 (67/67)	100 (93/93)	-	100 (81/81)	100 (79/79)	-
% Appropriate use (N 117)	75.44 (86/114)	67.39 (31/46)	0.32	75 (87/116)	68.18 (30/44)	0.42	81.32 (74/91)	62.32 (43/69)	0.01	71.43 (75/105)	76.36 (42/55)	0.57	71.64 (48/67)	74.19 (69/93)	0.72	64.20 (52/81)	82.28 (65/79)	0.01

VTI: volume to infuse in a certain time

### Programming intravenous medication

**Fig. 1:**
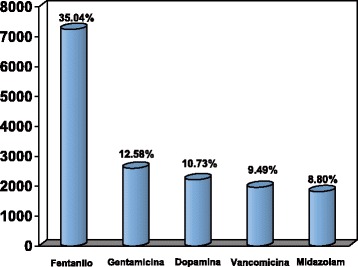
The most commonly used drugs in our unit in 2014. Fentanyl 35.04 % (7,246/20,680), Gentamicin 12.58 % (2,601/20,680), Dopamine 10.73 % (2,218/20,680), Vancomycin 9.49 % (1,962/20,680), and Midazolam 8.80 % (1,819/20,680)

Data was collected on the programming of 44,924 infusions of intravenous medication in 2014. In 46.03 % (20,680/44,924) of cases the name of the drug administered was recorded in the safety program. The drugs most commonly administered by intravenous infusion during the year are shown in Fig. 1. In 2.5 % (532/20,680) of the cases, there was an attempt to exceed the absolute limit when programming the infusion. This occurred in 4.46 % (323/7,246) of the programmed fentanyl infusions and in 11.21 % (204/1,819) of the programmed midazolam infusions. Together fentanyl and midazolam account for 99.05 % of the cases in which there was an attempt to exceed the absolute limit.

**Fig. 2:**
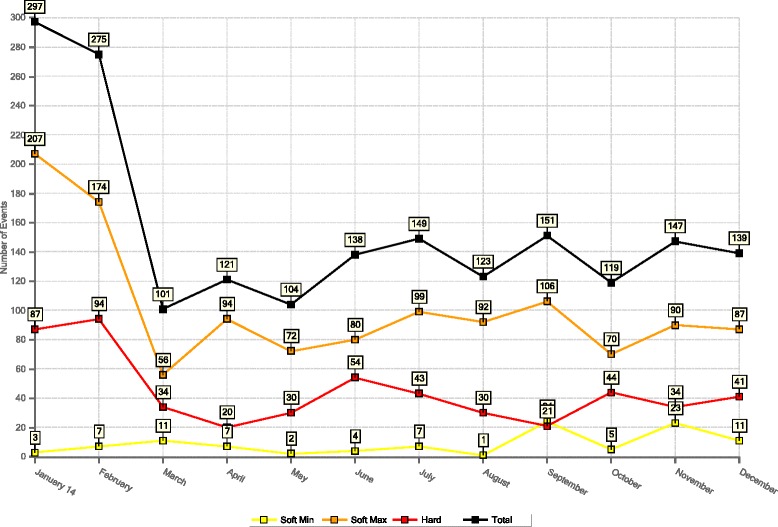
Distribution by month of alarms detected in medication programming in 2014

**Fig. 3:**
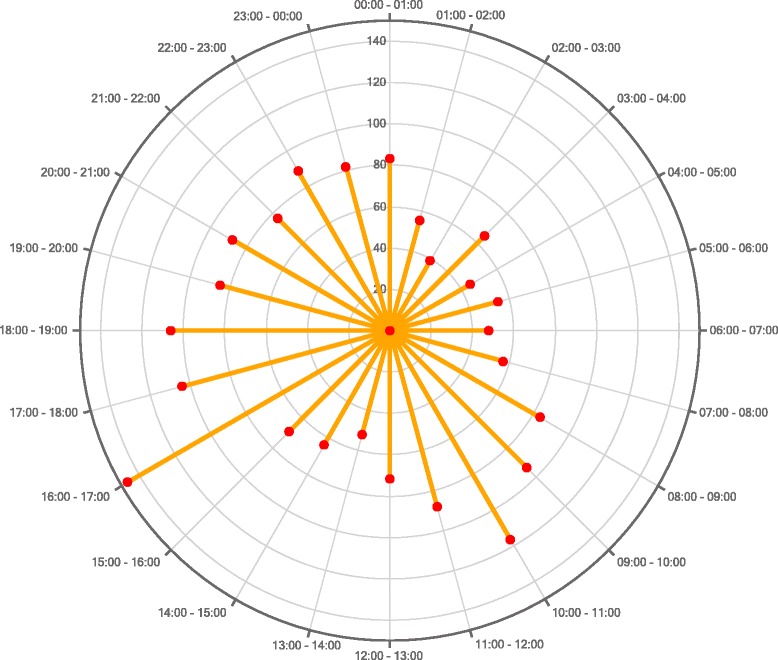
Distribution by time of day of alarms detected in medication programming in 2014

Figure 2 shows the monthly distribution of safety alarms in the medication programming, detected by the new software, and Fig. 3 shows the hourly distribution.

## Discussion

This study shows how the use of the infusion pumps before the intervention was not appropriate, especially in relation to the maximum infusion pressure limit. Changing the Alaris CC pump program to the Guardrails CQI Event Reporter® system and implementing the training sessions resulted in a very significant improvement in the appropriate use of the pumps and also prevented overdose errors. The best use of the pumps was essentially due to programming appropriately the maximum infusion pressure alarm, but other parameters were also significantly improved such as programming correctly the volume to infuse during a specific period of time, which has clinical relevance as an added safety measure. It is estimated that 30–60 % of administration errors for intravenous medication are related to using infusion pumps [5]. Despite the crucial role that the pumps have in administering medication in the neonatal population, the authors have not identified any previous study in which Random Safety Audits have been performed in a NICU to assess these aspects or which have assessed the usefulness of infusion pump safety systems in neonatology.

The RSA enabled detection of a generalised incorrect use of the pumps in the first study period. In most cases, this was due to the pressure limit being too high (120 mmHg) and not correctly adjusted by the personnel. The pressure will depend on different factors such as line type (central or peripheral), catheter type, or infusion rate. It seems that extravasation can occur during gravity infusions with pressure of around 70 mmHg. As such, the infusion pump manufacturers recommend individual setting of the pressure limit and programming it to 20–50 mmHg above the baseline pressure [11, 12].

After analysing all the data collected during the first period, and alarmed by the inappropriate use of the pumps, we designed different strategies to urgently improve this aspect of patient safety. A lower default pressure limit alarm was preset (60 mmHg), which could be modified by the nurse as appropriate.

The new Guardrails CQI Event Reporter® software, CareFusion, was installed on the pumps. It is a medication safety and quality auditing system specifically designed for infusions that presents a series of advantages. Firstly, the built-in drug library has more intuitive and simple programming. The doses, concentrations, and specific dose limits for the neonatal population were also updated in the drug library. Secondly, the new software allowed data on administration of medication and pump usage to be stored: the data recorded on the infusion devices is very useful to monitor compliance with the unit’s recommendations and protocols [5]. Infusion pumps with these built-in safety systems are known as smart infusion pumps [13, 14].

The data recorded during the year after introducing the new software showed the extremely large number of programmed infusions and, thus, the importance any measure that increases their safety can have. In one year, the installed safety program detected more than 500 attempts to program infusions with a dose above the present absolute safety limits. This means that the pump safety program was effective in preventing drug overdoses and their potential adverse effects in patients in a large number of cases, results which are similar to those of other studies [13–18]. Most attempts to exceed the absolute limits occurred with two drugs: fentanyl and midazolam; consequently, their protocols will have to be reviewed in order to identify why there is a tendency to program an excessive dose.

In our NICU, most alarms occurred around 10:00–11:00 a.m. and around 4:00–5:00 p.m., which coincide respectively with the time when the neonatologists change the treatment prescriptions and the period after the change in nursing shift, when the nurses start administering the medication. This differs from other studies, in which the time of greatest error coincides with the change in nursing shift or with other medication administration times [17, 19]. The months in which most alarms were detected due to attempts to exceed the relative and absolute limits in the programming of intravenous medication were January and February 2014, twice as many as in the other months of the year. We cannot explain this result since the NICU occupancy was practically the same throughout the year (96 %) and the introduction of the safety program and the personnel training had ended approximately 6 months earlier.

No relation between appropriate use of the pumps and patient characteristics has been found. However, the RSA performed during the second study period showed that pump use was better in the NICU small boxes. This is probably explained by the fact that perhaps there are fewer interruptions in the smaller areas, with fewer patients and less personnel, and this probably contributes to a higher concentration of staff when the pump safety systems are programmed.

Although the smart infusion pumps have the potential to reduce errors [20], their efficacy is often compromised in daily clinical practice by a lack of compliance with the recommendations/protocols for use, overlooking their safety alarm systems or ignoring alarms. Several studies show that one of the possible causes is nurses’ lack of experience, in particular those with less than 6 years’ experience [21]. They also show how nurse training effectively contributes to decreasing the frequency and severity of errors related to intravenous medication [21–23]. For this reason, theoretical sessions and practical workshops were given to doctors and nurses in our unit to improve the training in the use of these devices, thereby increasing awareness of the importance of complying with the protocols.

Our results show that the name of the drug administered was recorded in the safety program in 46.03 % of cases. However, we cannot interpret this result as adherence to the use of the program since in our unit the syringe pumps are also used for other infusions (saline solution or platelet transfusions) that are not included in the program’s drug library. As such, in the 54 % of cases in which a drug name was not recorded we cannot know in which cases it was due to an incorrect use of the safety program because the drug was in the library or in which cases the infusion was a saline bolus or other medication that is not in the library and so no name could be recorded.

During the second study period, audits were performed for only three months as, out of the 23 technological equipment/procedures included in the initial study, only the infusion pumps were being audited and because the results obtained showed a large improvement compared to the first period.

Although this use of RSAs may be surprising, as they have traditionally been used for monitoring, as mentioned in the introduction, they can also be used to consistently detect unknown weakness which, once identified, could lead to interventions. These procedures can continue to be monitored after the intervention. Error identification by audits maintained over time can identify repeated weaknesses in the system that do not depend on either the professionals working at any given time or on the circumstances.

One of the limitations of this study is that the extravasations of the lines that occurred during the study periods were not recorded; this would have enabled the clinical impact of better programming of the pump infusion pressure limit to be known. Also audits were not performed during the night shift. In the first period, as previously noted, all the days when the pumps were audited were (by chance) working days. In the second period, working days and weekends/holidays were studied, and no differences in use were identified.

## Conclusion

The Random Safety Audits were a very useful tool for detecting inappropriate use of the pumps in a Neonatal Intensive Care Unit. Introducing safety software on the infusion pumps, preparing a written protocol for use, and training sessions were effective strategies for improving appropriate use of the infusion pumps for intravenous medication in our NICU.

## Abbreviations

RSA: random safety audits
NICUs: Neonatal Intensive Care Units
CI: confidence intervals
VTI: volume to infuse in a certain time
